# The complete chloroplast of *Chrysanthemum × morifolium* ‘Fubaiju’

**DOI:** 10.1080/23802359.2021.1960213

**Published:** 2021-09-27

**Authors:** Tuo Zeng, Jiawen Li, Qiang Fu, Caiyun Wang

**Affiliations:** a Key Laboratory for Biology of Horticultural Plants, Ministry of Education, College of Horticulture & Forestry Sciences, Huazhong Agricultural University, Wuhan, China; b School of Life Sciences, Guizhou Normal University, Guiyang, China

**Keywords:** Asteraceae, *Chrysanthemum × morifolium*, chloroplast genome, phylogenetic analysis

## Abstract

*Chrysanthemum × morifolium* ‘Fubaiju,’ which is native to Macheng, Hubei, China, has a long cultivation history almost dating back to the early 10th century Song dynasty, and is used as Chrysanthemum tea drink and Chinese traditional medicine. In this study, the complete chloroplast genome sequence of ‘Fubaiju’ was 151,109 bp, included a large single copy LSC (82,931 bp), a small single copy SSC (18,350 bp), and a pair of inverted repeat regions (24,941 bp). It contained 132 genes with 87 CDS, 8r RNA, and 37 tRNA. The phylogenetic analysis showed that the *C. × morifolium* ‘Fubaiju’ was clustered together with *C. × morifolium* ‘Baekma’.

*Chrysanthemum × morifolium*, belongs to the family Asteraceae, is one of the top ten famous traditional flowers in China, has been widely used in ornamental. Some of the cultivars also have other functions, such as anti-inflammatory, antioxidant, prevent colds, enhanced eyesight, and regulate immunity (Bensky and Stöger 2004; Han et al. 2019; Wang et al. 2020), and widely used in Chinese traditional medicine and edible (Sun et al. 2015).

The *C. × morifolium* ‘Fubaiju’ as a product of geographic indications in China originated in Macheng city, was mainly used for tea drink. However, the taste and the quality of *Chrysanthemum* tea have significantly depended on the *Chrysanthemum* cultivars, and it is hard to identify from appearance. Therefore, it needs rapid and simple methods to determine the *C. × morifolium* ‘Fubaiju.’ The chloroplast genomes were widely used as DNA barcodes to identify plants (CBOL Plant Working Group. 2009) . Therefore, using the chloroplast genomes of *C. × morifolium* was useful for the identification of cultivars and phylogenetic studies.

*Chrysanthemum × morifolium* ‘Fubaiju’ was collected from Macheng city Hubei province (31°19′55.48ʺN, 115°05′88.73ʺE), and transferred to flower nursery stock of Huazhong agricultural university, the specimen was preserved in the Museum of Huazhong Agricultural University(http://bwg.hzau.edu.cn/, Fu Qiang fuqiang@mail.hzau.edu.cn under the voucher number ccau0013597), the fresh leaves of *C. × morifolium* ‘Fubaiju’ were selected and the genomic DNA was extracted using Plant Genomic DNA Kit (Tiangen China), the whole chloroplast genome sequences was analysis on Illumina HiSeq 2500 by Genesky Biotechnologies Inc. (Shanghai, China) and assembled by metaSPAdes (Nurk et al. 2017) and NOVOPlasty (Dierckxsens et al. 2017), with the reference genomes of *C. × morifolium* NC020092 and *C. lucidum* NC_040920, the annotation was performed using Geseq (Tillich et al. 2017) and CPGAVAS2 (Shi et al. 2019) and checked by manual inspection. submitted to NCBI GenBank under the accession number MT919691 (*C. × morifolium* ‘Fubaiju’).

The *C. × morifolium* ‘Fubaiju’ chloroplast genome is 151,109 bp with a typical quadripartite, and conservative structure including a large single copy (LSC) regions of 82,931 bp, a small single copy(SSC) of 18,350 bp, a pair of 24,941 bp of inverted repeat regions, the GC content of the cp genome is 37.5%, the cp genome contains 132 genes include 87 CDS, 8 rRNA and 37 tRNA.

To reveal its taxonomic location, 67 common protein-coding sequences from the cp genomes of 17 species were selected and using *Ismelia carinata* as the outgroup. The CDS were aligned by maftt v7 (Katoh and Standley 2013), respectively. After aligned, these sequences were concatenated by phylosuite v1.2.2 (Zhang et al. 2020), and the maximum-likelihood (ML) phylogenetic tree was constructed by IQtree2 (Minh et al. 2020), with the TVM + F+R3 model selected by ModelFinder (Kalyaanamoorthy et al. 2017) and 1000 bootstrap replications (Figure 1) . The results of the ML trees shown that the *C. × morifolium* ‘Fubaiju’ was clustered together with *C. × morifolium* ‘Baekma’ (MK 986830). This cp sequence of *C. × morifolium* ‘Fubaiju’ can be helpful for cultivar identification, DNA barcode, and phylogenetic analysis.

**Figure 1.**
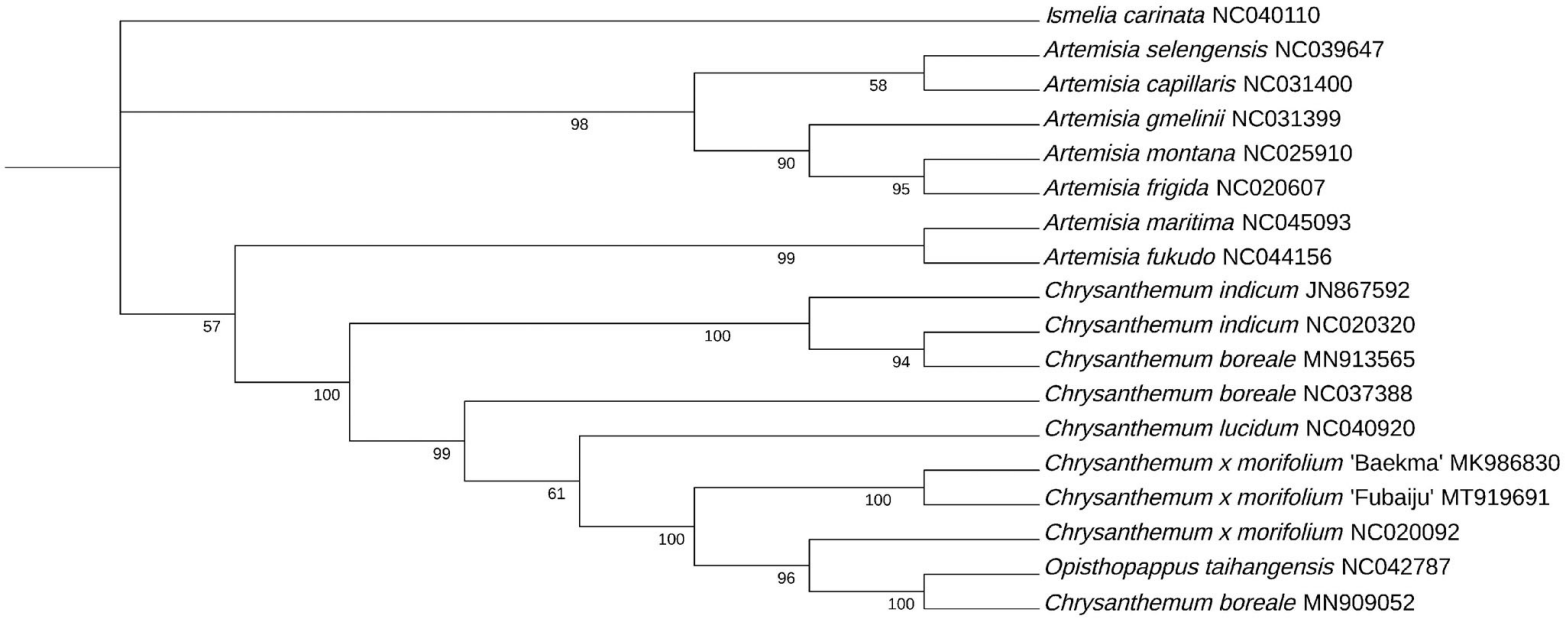
Phylogenetic tree constructed by the maximum-likelihood (ML) method with 1000 bootstrap replicates, the bootstrap support values are shown at the branches. GenBank accession numbers were also shown.

## Data Availability

The genome sequence data that support the findings of this study are openly available in GenBank of NCBI at (https://www.ncbi.nlm.nih.gov/nuccore/MT919691.1) under the accession no. MT919691.1. The associated BioProject, SRA, and Bio-Sample numbers are PRJNA738294, SRR14826271, and SAMN19717678 respectively.
